# Analytical performance of the selective, automatic multianalyser Olympus AU 5031

**DOI:** 10.1155/S1463924690000025

**Published:** 1990

**Authors:** C. Luley, H. G. Struss, W. Prellwitz

**Affiliations:** 1 Abteilung für Klinische Chemie und Laboratoriumsmedizin Universitätsklinik der Johannes-Gutenberg- Universität Mainz Germany; 2 Zentrallabor der Universitätsklinik Hugstetter Strasse 55 Freiburg 7800 Germany

## Abstract

The analytical performance of the selective, automatic multianalyser Olympus AU 5031 was evaluated over four months and assessed for practicability for another eight months. The evaluation followed the ECCLS guidelines. Twenty routine parameters were measured. In addition, sodium and potassium were determined on an attached flame photometric unit. Both the agreement between the eights photometers per unit and the temperature behaviour in the cuvettes was satisfactory. The imprecisions were very good. The within-run imprecision was below 1.5% for the majority of the parameters. The imprecision between days was below 5%, with the exception of creatine phosphokinase (7.4%). Glutamate dehydrogenase gave an imprecision of between 4.0% and 15.9%, which, however, is more likely due to the low activities measured rather than the fault of analyser. The recovery of the assigned values in 12 control sera was between 95% and 105% for 14 tests. Three of the remaining eight tests yielded recoveries with deviations between 10% and 18% (alanine aminotransferase, aspartate aminotransferase and bilirubin). No drift effects were observed and neither a sample carry-over nor a reagent carry-over were detected. Most tests were linear over a very wide range. Only afew tests (mainly lipase and glutamate dehydrogenase) required measurement repetitions with diluted samples. The correlation with routine instruments and tests was close. However, corrections were necessary for 14 of the 22 tests. This was not due to the performance of the analyser but, rather, to the different methodologies of compared tests, or different working temperatures on the comparison instruments, or a lack of accuracy for some of the AU tests.


Journal of Automatic Chemistry Vol. 12, No. 1 (January–February 1990), pp. 2–16

### Analytical performance of the selective, automatic multianalyser Olympus AU 5031

**C. Luley†, H. G. Struss and W. Prellwitz**

*Abteilung für Klinische Chemie und Laboratoriumsmedizin, Universitätsklinik der Johannes-Gutenberg-Universität, Mainz, FR Germany*

*The analytical performance of the selective, automatic multianalyser Olympus AU 5031 was evaluated over four months and assessed for practicability for another eight months. The evaluation followed the ECCLS guidelines. Twenty routine parameters were measured. In addition, sodium and potassium were determined on an attached flame photometric unit. Both the agreement between the eights photometers per unit and the temperature behaviour in the cuvettes was satisfactory. The imprecisions were very good. The within-run imprecision was below 1·5% for the majority of the parameters. The imprecision between days was below 5%, with the exception of creatine phosphokinase (7·4%). Glutamate dehydrogenase gave an imprecision of between 4·0% and 15·9%, which, however, is more likely due to the low activities measured rather than the fault of analyser. The recovery of the assigned values in 12 control sera was between 95% and 105% for 14 tests. Three of the remaining eight tests yielded recoveries with deviations between 10% and 18% (alanine aminotransferase, aspartate aminotransferase and bilirubin). No drift effects were observed and neither a sample carry-over nor a reagent carry-over were detected. Most tests were linear over a very wide range. Only a few tests (mainly lipase and glutamate dehydrogenase) required measurement repetitions with diluted samples. The correlation with routine instruments and tests was close. However, corrections were necessary for 14 of the 22 tests. This was not due to the performance of the analyser but, rather, to the different methodologies of compared tests, or different working temperatures on the comparison instruments, or a lack of accuracy for some of the AU tests.*

### Introduction

The Olympus AU is an analyser for medium and large laboratories. This paper reports on the performance of the AU 5031. The evaluation lasted six months and the instrument has been in routine use for a further eight months. Although the authors accept that only a multicentre evaluation, as suggested by ECCLS guidelines [1], allows a truly representative assessment for an analyser, multicentre evaluations of analysers of this size require considerable time. The evaluation data are reported here because there is a growing interest in the performance characteristics of the Olympus 5000.

The protocol of this evaluation followed the ECCLS guidelines; changes were made only if they seemed appropriate to the specific features of the AU analyser.

† To whom correspondence should be addressed. Present address: Zentrallabor der Universitätsklinik, Hugstetter Strasse 55, 7800 Freiburg, FR Germany.

Recently, two evaluations of this analyser have been published [1 and 2]. They, too, followed the ECCLS guidelines but some aspects reported here were not studied or were approached differently. Therefore, the present data and that in the previous reports may serve as a preliminary multicentre evaluation.

### General description of the AU 5000 analyser

The AU 5000 Analyser is constructed according to a modular concept. Any analyser consists of up to eight units. Each unit is basically a complete analyser and is equipped with either four, eight or 12 reagent lines. The specifications of such a unit are given in table 1. By combining several units the performance of the resulting analyser can be tailored to the requirements of the user in respect to sample throughput, number of tests or both. By choosing the maximum number of 12 reagent lines, the user gains a higher number of available tests at the cost of sample throughput. Possible combinations of available test numbers and throughput capacities are displayed in table 2. However, once a certain configuration is installed it must remain permanent.

In cases of malfunction of a unit, it can be exchanged in less than an hour. Consequently, any AU analyser which contains more than one unit, can, to a certain degree, supply its own back-up system. However, some parts of the analyser have a common function, and, in the event of failure, will cause the analyser to stop. The most important of these is the sample rack transport system and the data-processing unit.

The AU configuration which was evaluated, an AU 5031, consists of three units with eight reagent lines per unit. It processes 150 samples per hour and with an additional (optional) flame photometer carries out 26 tests per sample. Twenty-four cuvettes are assigned to each test and complete one cycle of the cuvette wheel in 8 min, and 24 s. If a test is not required for a given sample, the cuvette passes empty and is washed. Consequently, the processing speed remains constant regardless of the number of tests required for a given sample.

Most of the reagents kits for the AU 5000 were from the Merck Company in Darmstadt, FR Germany. However, there are other suppliers who offer tests which are tailored to this analyser. In addition, since this system is entirely open, many commercial tests can be adapted to the analyser.

0142–0453/90 $3.00 © 1990 Taylor & Francis Ltd.

*Table 1. Instrument specifications for the AU 5031.*

| | |
|---|---|
| 1. Type of instrument | Discrete selective, automatic multianalyser. |
| 2. Test procedures | End-point, endpoint with sample blank, kinetic with and without sample start, linear and non-linear calibration, serum indices (haemolysis, lipaemia, hyperbilirubinaemia). |
| 3. Specimen carrier | Straight racks with 10 positions for patient's specimen (white), control sera (green), calibrators (yellow), water and reagent blanks (blue) and emergency samples (Stats) (red). Specimen identification via bar-code reader. |
| 4. Sampling system | Pipettors in flame unit and each module with two liquid level sensors driven by stepping motors. |
| 5. Sampling volumes | 3–15 μl in 1 μl adjustable steps. Flame 50 μl. |
| 6. Sample dispensing | Four tests – divided dispensing by means of micro syringe, constant volume pump. |
| 7. Urgent samples | Preferential processing of Stats possible. |
| 8. Reagent storage | Refrigerated storage (5°–15 °C). Ambient temperature storage compartment also provided. |
| 9. Reagent dispensor | Two independent pipettors per method. |
| 10. Reagent volumes | Reagent 1 and 2: 50–500 μl. |
| 11. Reaction rotor | Turntable with 192 cuvettes. |
| 12. Reaction cuvettes | 192 quartz glass cuvettes. 6 mm × 5 mm × 30 mm Pyrex glass cuvettes. Minimum volume 250 μl; pathlength 6 mm. |
| 13. Reaction time | 8 min 24 s. 2nd reagent is dispensed after 3 min 36 s. |
| 14. Reaction temperature | 37 °C +/− 0·2 °C in a dry bath with incubation fluid circulation system. |
| 15. Light source | Halogen lamp 12 V, 100 W. |
| 16. Photometry system | Direct photometry in the reaction vessel. Measuring OD: 0·0–2·0. One or two wavelengths, photodiode, 8-point photometry. |
| 17. Wavelength | Interference filter: 340, 380, 410, 480, 520, 540, 570, 600, 660, 750 and 850 nm. |
| 18. Reaction solution | Through the rotating action of the stirring bar after the second reagent is dispensed. |
| 19. Sample cup | 16 × 100 mm, polystyrene tube. |
| 20. Test item selection | Single, panels of tests or combined selection possible, definition of up to 10 panels. |
| 21. Data memory capacity | Routine samples: 4000. Emergency samples 200. |
| 22. Quality control | Daily and day-to-day mean, range, standard deviation and coefficient of variation calculations and graphs. |
| 23. Data processing | Correlation correction (AX + B), sample blank correction, data correction for each chemistry (AX + B), serum quality judgement, abnormal upper/lower value judgement, calculation of test ratios, work list, abnormal value/pending test list, test result editing for each sample. |
| 24. Water supply | Continuous flow, deionized with shut-off valve. |
| 25. Water consumption | 15 l/analyser unit/h. |
| 26. Waste drainage | Separately expelled: chemical and non-hazardous waste. |

### Methods

*Agreement between photometer and temperature behaviour in cuvettes*

Two hardware conditions were investigated: the linearity in all eight photometers in a unit and the temperature behaviour in a cuvette after addition of cooled (8 °C) reagent 1.

For the linearity experiment, two solutions were prepared with NADH and para-nitrophenol, respectively, which covered in seven dilution steps the extinction range between 0·5 and 3·0 for each solution. The extinctions of each solution were measured in triplicate and were plotted in eight parallel extinction curves for visual inspection of linearity and parallelity.

The temperature in the cuvette was measured with a micro temperature sensor in a cuvette to which 10 μl of sample and, 12 s later, 500 μl of reagent 1 was added. In order to ensure that cooled reagent 1 (8 °C) reached the cuvette, the reagent was dispensed repeatedly immediately before the experiment. The rationale of this experiment was to check whether the temperature in the cuvette reached 37 °C before reading by photometer 1, 168 s later.

*Table 2. Analyser configurations and throughput.*

| | | | | | | |
|---|---|---|---|---|---|---|
| Number of units | 1 | 2 | 3 | 4 | 6 | 8 |
| Number of reagent lines per unit | 12 | 12/8 | 8/4 | 8/4 | 4 | 4 |
| Available tests per analyser | 24 | 24/16 | 24/12 | 32/16 | 24 | 32 |
| Sample processing capacity (per h) | 50 | 100/150 | 150/300 | 150/380 | 300 | 300 |

Example: An Olympus analyser of the 5030 series consists of three units each of which will be equipped with either eight or four reagent lines. This will, for the total unit, yield 12 or 24 available tests, respectively, allowing a throughput of 150 or 300 samples/h, respectively.

*Table 3. Reagents used during the evaluation.*

| Analyte | Method | Manufacturer |
|---|---|---|
| *1. Enzymes* | | |
| Alkaline | 'Opt. stand. method'† | Merck 14 000 |
| Alanine aminotransferase | Opt. UV-test; modified | Merck IFCC method 14 344 |
| Amylase | Substrate: 2-Cl-PNP- | Merck β,D-maltoheptaosid 14 159/158 |
| Aspartate aminotransferase | Opt. UV-test; modified | Merck IFCC method 14 346 |
| Creatine phosphokinase | 'Opt. stand. method' | Merck 14 109/111 |
| γ-glutamyl transferase (new) | 'Szasz' | Merck 14 056/057 |
| Glutamate dehydrogenase | 'Opt. stand. method' | Boehringer 124 320 |
| Lactate dehydrogenase | Opt. stand. method' | Merck 3399 |
| Lipase | Turbidimetry substrate | Boehringer Triolein 262 358 |
| *2. Flame photometry* | | |
| Sodium | With flame photometer | Merck Lithium as internal standard |
| Potassium | With flame photometer | Merck Lithium as internal standard |
| *3. Substrates* | | |
| Blood urea nitrogen | Urease/glutamate dehydrogenase | Merck 14 135/137/161 |
| Bilirubin | 2,5-Dichlorophenyl-diazonium salt (DPD) | Merck 14 162/163 |
| Calcium | ortho-cresol-phthalein-complexon | Merck 19 724 |
| Cholesterol | Cholesterol oxidase/peroxidase (CHOD-PAP) | Merck 14 138/139/140/141 |
| Creatinine | Modified Jaffé without deproteinization | Merck 19 726/727 |
| Glucose | Glucose dehydrogenase without deproteinization | Merck 14 051/055 |
| Phosphate | Molybdate reaction | Merck 19 723 |
| Iron | Ferrozin method | Merck 19 725 |
| Magnesium | Xylidylblue | Merck 19 736 |
| Total protein | Biuret reaction | Merck 3327 |
| Triglycerides | Enzymatic UV-test | Merck 14 151/148 |
| Uric acid | Uricase/peroxidase | Merck 14 126/27/28 |

† Optimized standard method according the Deutsche Gesellschaft für Klinische Chemie.
NN: not yet in the catalogue.

*Reagents and comparison instruments*

The reagents used in this evaluation are listed in table 3. The comparison instrument and the reagents used in the comparison instrument are given in table 4. While some comparison procedures were virtully identical (in particular most enzymes), the following were different in either chemistry or test principle: amylase, BUN, bilirubin, calcium, magnesium and uric acid.. In addition, there were differences in measurement temperatures: while the AU 5031 works at 37 °C some of the comparison procedures operated at either 25 °C (amylase, lipase, lactate dehydrogenase, BUN, cholesterol, creatinine,

*Table 4. Analytical instruments and tests used for method comparison.*

| Analyte | Instrument | Method | Reagents |
|---|---|---|---|
| *1. Enzymes* | | | |
| Alkaline phosphatase | ERIS[1] | 'Opt. stand. method' | Merck 19 711 |
| Alanine aminotransferase | ERIS[1] | Opt. UV-test; modified IFCC method | Merck 19 709 |
| Amylase | COBAS BIO[2] | Substrate: Maltotetraose | Gödecke 847 899 |
| Aspartate aminotransferase | ERIS[1] | Opt. UV-test; modified IFCC method | Merck 19 708 |
| Creatine phosphokinase | ERIS[1] | 'Opt. stand. method' | Merck 19 715 |
| γ-glutamyl transferase (new) | ERIS[1] | 'Szasz' | Merck 19 710 |
| Glutamate dehydrogenase | ERIS[1] | 'Opt. stand. method' | Merck 19 734 |
| Lactate dehydrogenase | COBAS BIO[2] | 'Opt. stand. method' | Merck 3399 |
| Lipase | HITACHI 705[3] | UV-test; substrate: Triolein | Boehringer 262 358 |
| *2. Flame photometry* | | | |
| Sodium | FL 7[4] | With flame photometer lithium as internal standard | |
| Potassium | FL 7[4] | With flame photometer lithium as internal standard | |
| *3. Substrates* | | | |
| Blood urea nitrogen | SMA[5] | Diacetyl monoxime method | Technicon T40-0001 |
| Bilirubin | COBAS BIO[6] | Jendrassik Grof reaction | Hoffmann la Roche 07 100 32 |
| Calcium | FL 7[7] | Atom absorption | |
| Cholesterol | COBAS BIO[2] | Cholesterol oxidase/peroxidase (CHOD-PAP) | Boehringer 692 905 |
| Creatinine | SMA[5] | Jaffé | Technicon T40-0004 |
| Phosphate | SMA[5] | Molybdate reaction | Technicon T40-0012 |
| Iron | COBAS BIO[6] | Ferrozin method | Hoffman la Roche 07 105 98 |
| Magnesium | FL7 | Atom absorption | |
| Total protein | COBAS BIO[6] | Biuret | Hoffman la Roche 07 100 83 |
| Triglycerides | COBAS BIO[6] | Enzymatic UV-test | Merck 14 360 |
| Uric acid | SMA[5] | Sodium tungstate method | Technicon T40-0014-00 |

[1] ERIS: Eppendorf Gerätebau Netheler + Hinz GmbH, Hamburg, FR Germany.

[2] COBAS BIO: Hoffmann la Roche & Co. AG, Basel, Switzerland.

[3] HITACHI: Boehringer, Mannheim, FR Germany.

[4] FL 7: Zeiss GmbH, Oberkochem, FR Germany.

[5] SMA: Technicon GmbH, Bad Vilbel, FR Germany.

[6] COBAS BIO: Hoffmann la Roche & Co. AG, Basel, Switzerland.

[7] FL 7: Zeiss GmbH, Oberkochem, FR Germany.

phosphate, uric acid) or 30 °C (bilirubin). All enzyme activities which were measured on the AU 5031 at 37 °C were converted to corresponding activities at 25 °C.

*Imprecision*

The within-run imprecision was determined from 20 measurements of three commercial control sera. Care was taken to include a control serum containing normal analyte levels (Monitrol I). The series were repeated three times and the medians of the three CV values were taken as the final results.

The same sera were used for the determination of between-day imprecision, which was determined over 21 days (18 working days).

Predetermined factors were used for the measurement of enzyme activities. Substrate and electrolyte concentrations were calibrated every day with the same calibrator ('Calibrator for automated systems', Boehringer Mannheim GmbH, Catalogue No. 759 350). The control sera used in this experiment are listed in table 5.

*Drift*

Drift effects were studied using three commercial and control sera covering different levels of the analytes measured. The sera were dissolved in the morning and split into nine aliquots which were sealed and kept at 4 °C until use. Measurements of aliquots were carried out every 60 min for 8 h. For determination of alkaline phosphase and bilirubin the sera were freshly reconstituted every 2 h.

*Range limits*

Linear ranges were investigated for all tests either by diluting very high concentrations or activities, respectively, of the analyte, or by spiking human serum with the pure analyte. The measured concentrations were plotted against dilutions and the resulting graphs were inspected visually for linearity.

*Carry-over*

Carry-over effects are limited due to the construction principle that each cuvette is used for one test only. However, carry-over might occur as sample carry-over due to insufficient cuvette washing (specimen-related carry-over), through inadequate cleaning of the mixer blades (specimen-independent carry-over).

*Specimen-related carry-over*

Sample carry-over was tested by determining the activity of alkaline phosphatase during two complete cuvette wheel cycles ($N = 48$ samples). All samples were taken from a serum pool with a low activity of 63 U/l, except sample numbers 6 to 10 and 16 to 20 which contained the very high activity of 5200 U/l. Thus, during the second cuvette wheel cycle (sample numbers 25 to 48) samples containing the low activity were measured in cuvettes which were used for the determination of a very high activity of the analyte in the previous cuvette wheel cycle. If the cuvettes are washed, residue of the sample with high alkaline phosphatase activity should cause elevated results in cuvette numbers 6 to 10 and 16 to 20, respectively.

*Specimen-independent carry-over*

In order to investigate the potential reagent carry-over by mixer blades, the combination of lipase and triglyceride determinations was chosen. The first reagent line in a single unit was used for lipase assay and the fifth reagent line for the triglyceride test. Since samples were processed in groups of four, the first of the four mixer blades stirred first the lipase reaction mixture in cuvette 1 and subsequently (after the mixer blade washing procedure) the reaction mixture for the triglyceride determination in cuvette 5.

This should lead to falsely high measurements of triglyceride if reaction solution was transferred from cuvette (lipase assay) to cuvette 5 (triglyceride assay). If the triglyceride determination only was required, the prewashed mixer blade just stirred the triglyceride reaction solutions in cuvette numbers $5 + 8N$ ($0 < N < 24$) in this unit. Thus, a potential carry-over from cuvettes $1 + 8N$ to

*Table 5. Control sera used for the AU 5031 evaluation*

| Control serum | Lot No. | Manufacturer Control | Serum in figures 1 and 2 |
|---|---|---|---|
| N + D Moni-trol I† | 208 | AHS/Deutschland GmbH, | + |
| M + D Moni-trol II† | 108 | Bereich Merz + Dade | + |
| Validate N† | 4 × 023 | Gödecke AG, | |
| Validate A | 4 × 625 | Freiburg, FRG | + |
| Seronorm | 174 | E. Merck AG, | |
| Pathonorm L | 20 | Darmstadt, FRG | |
| Pathonorm H | 21 | | |
| Precipath E | 152147 | Boehringer | |
| Precipath U | 151464 | Mannheim GmbH, | |
| Precinorm U | 154290 | FRG | |
| Kontrollogen-L | 623125 B | Behringwerke | |
| Kontrollogen-LP | 623211 H | AG, Marburg/Lahn | |
| | | FRG | |

† Used for determination of imprecision; all control sera were used in the recovery study.

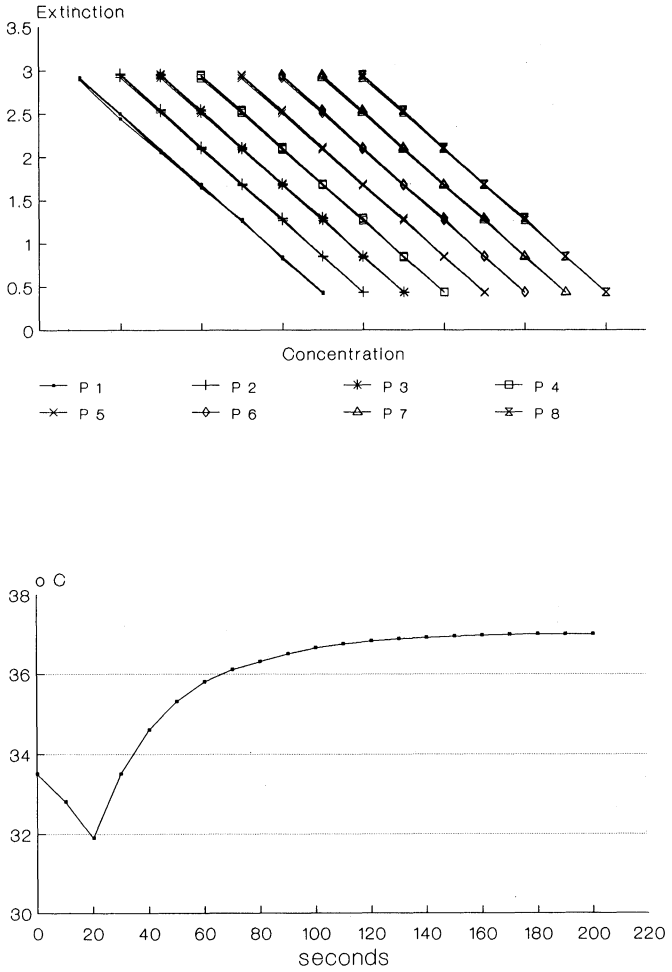

*Figure 1. Linearity and reproducibility of all eight photometers in a unit as measured by dilutions of paranitrophenol in triplicate (top). The temperature course in a cuvette upon addition of precooled (8 °C) is displayed below.*

cuvettes 5 + 8$N$ could be investigated by measuring triglycerides with and without determining lipase activity. This was carried out by determining the triglyceride levels in quadruplicate in 10 human serum samples, covering triglyceride levels between 0·4 and 4·3 mmol/l, with and without simultaneous lipase determinations. The triglyceride values of both groups (with and without lipase measurement) were compared by means of the Wilcoxon test.

*Assessment of accuracy*

*Recovery of assigned values in quality-control sera*

Twelve commercial sera had been selected for the determination of the recovery of assigned values in control sera (see table 5).

*Comparison with other analytical routine procedures*

One hundred fresh human sera from daily routine samples were determined in the AU 5031 analyser and in the comparison instrument. Care was taken to ensure simultaneous measurement of both instruments and to include pathological analyte levels.

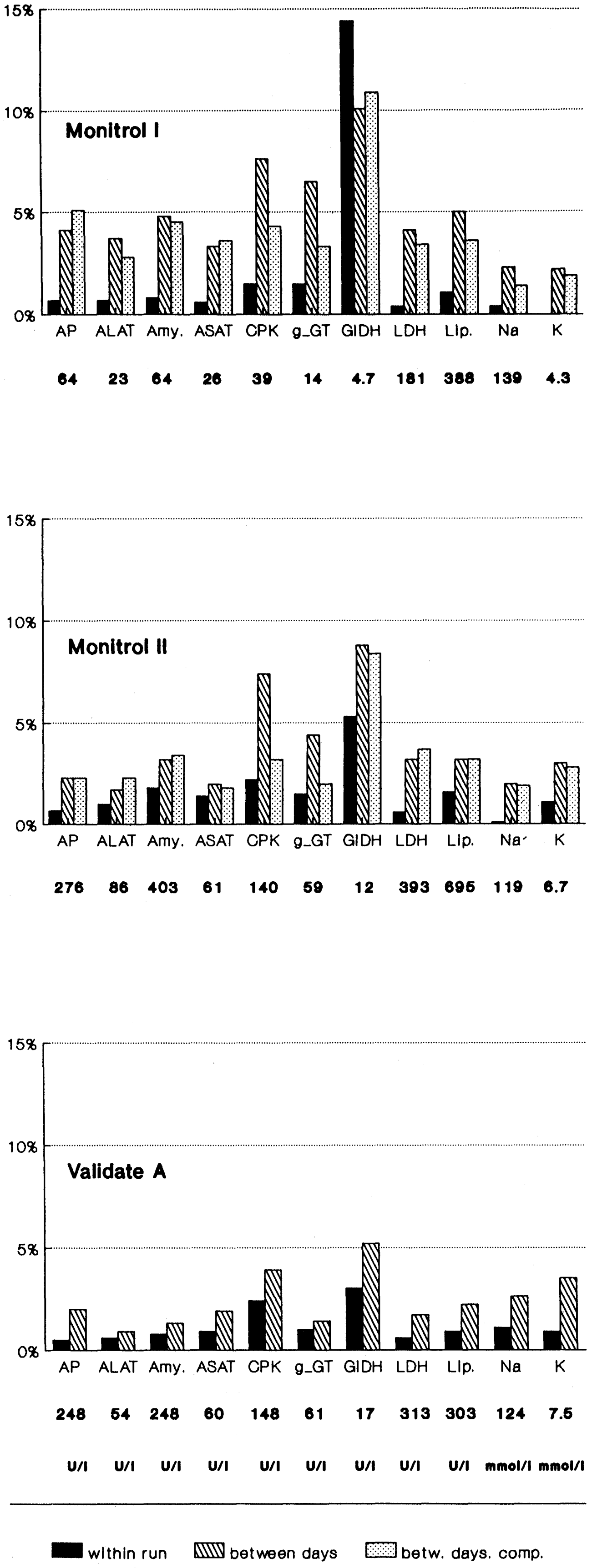

*Figure 2(a). Imprecision (enzymes and flame photometry): within-run and between-days on both AU 5031 and the respective comparison instruments. The assigned values in the three commercial control sera are given at the bottom of the graphs.*

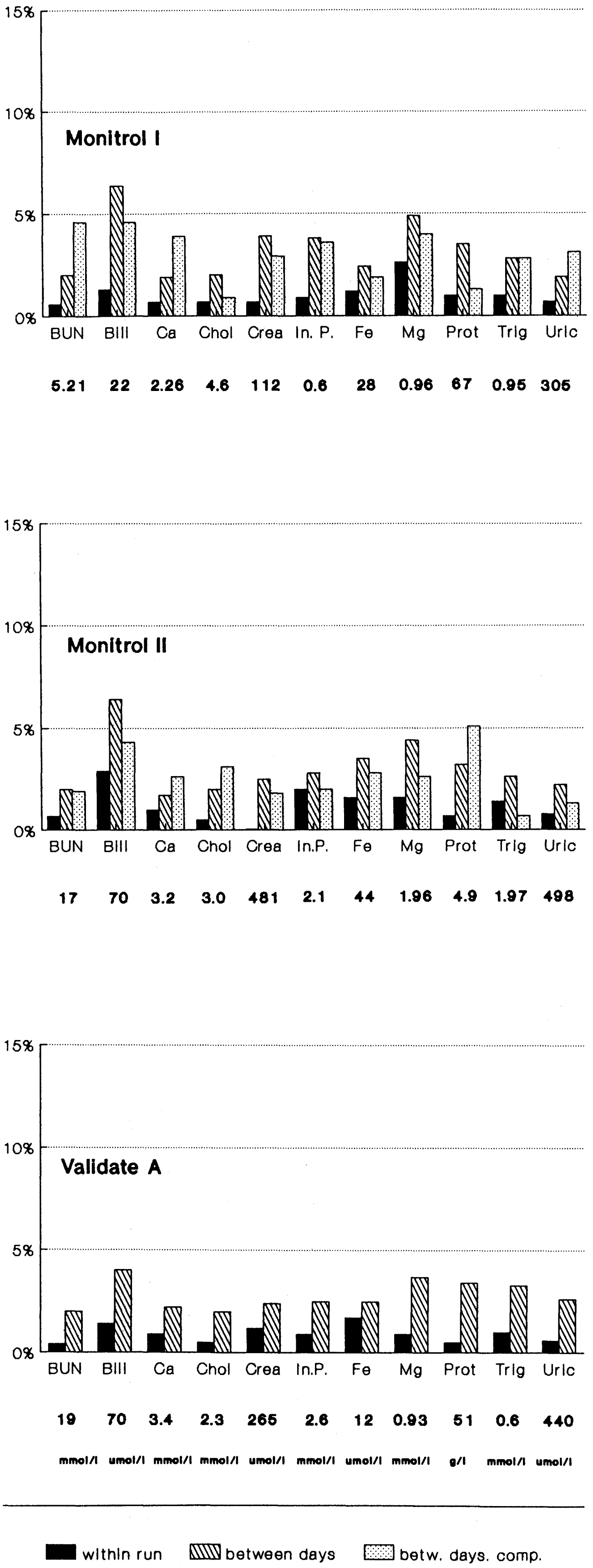

*Figure 2(b). Imprecison (substrates): within-run and between-days on both AU 5031 and the respective comparison instruments. The assigned values in the three commercial control sera are given at the bottom of the graphs.*

### Results

*Linearity of all eight photometers in a unit and temperature behaviour in cuvettes*

The extinction readings of a series of para-nitrophenol dilutions were linear, parallel and reproducible for each of the eight photometers (see figure 1, top). The time

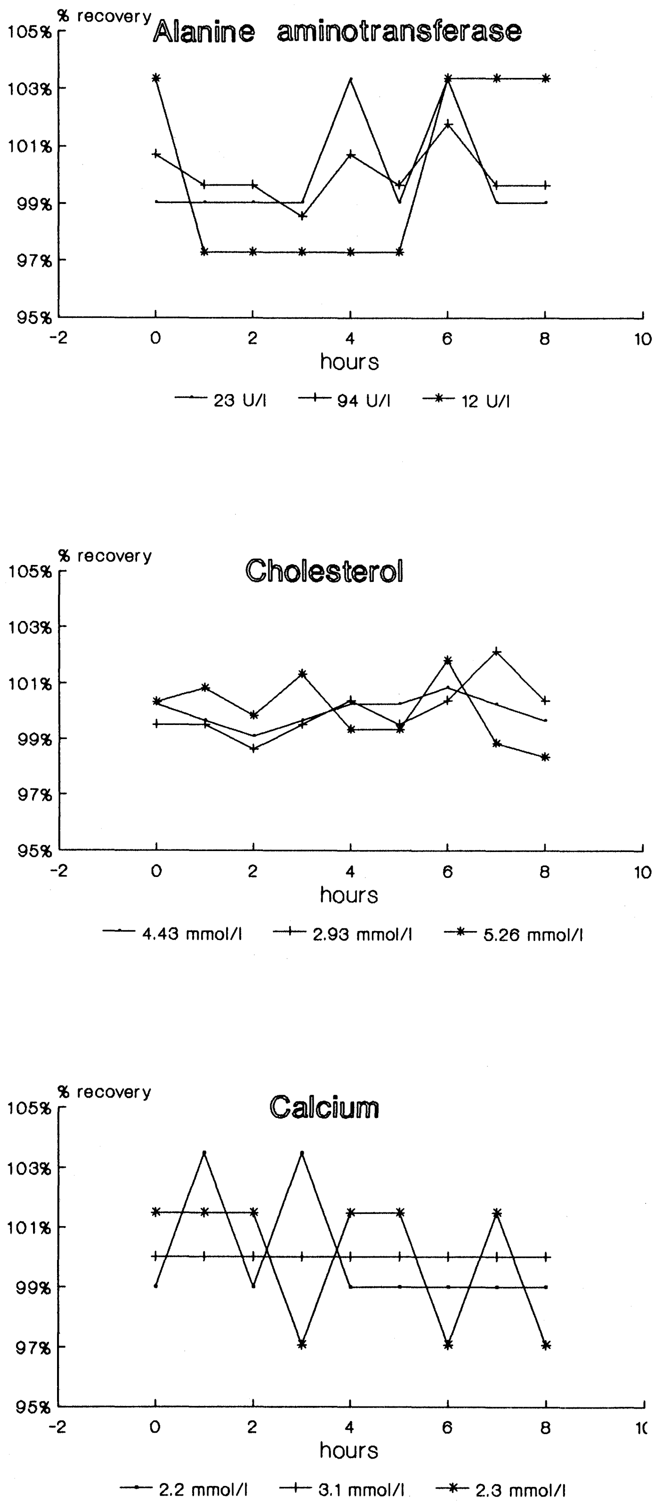

*Figure 3. Drift during 8 h for three representative parameters. The analyte activities and concentrations, respectively, are given at the bottom of the graphs.*

*Table 6. Assigned and found values in three commercial control sera. Between-day imprecision within-run imprecision. Three independent series and median.*

| | | | | Imprecision | | | | |
|---|---|---|---|---|---|---|---|---|
| | | | | | Within-run | | | |
| Analyte (Units) | Control material | Assigned values | Found value | Between days | (Three series) | | | Median |
| *1. Enzymes* | | | | | | | | |
| Alkaline phosphatase | M-I | 64 | 63 | 4·1 | 1·1 | 0·4 | 0·7 | 0·7 |
| (U/l) | M-II | 276 | 291 | 2·3 | 0·7 | 0·7 | 1·1 | 0·7 |
| | Val-A | 248 | 237 | 2·0 | 0·6 | 0·5 | 0·5 | 0·5 |
| Alanine aminotransferase | M-I | 23 | 17 | 3·7 | 0·6 | 0·7 | 0·7 | 0·7 |
| (U/l) | M-II | 86 | 64 | 1·7 | 0·9 | 1·0 | 1·9 | 1·0 |
| | Val-A | 54 | 44 | 0·9 | 1·0 | 0·6 | 0·5 | 0·6 |
| Amylase | M-I | 64 | 58 | 4·8 | 0·7 | 1·3 | 0·8 | 0·8 |
| (U/l) | M-II | 403 | 412 | 3·2 | 2·3 | 2·0 | 1·8 | 1·8 |
| | Val-A | 248 | 275 | 1·3 | 0·8 | 5·3 | 0·7 | 0·8 |
| Aspartate aminotransferase | M-I | 26 | 21 | 3·3 | 0·0 | 0·6 | 0·8 | 0·6 |
| (U/l) | M-II | 61 | 52 | 2·0 | 1·9 | 1·4 | 1·4 | 1·4 |
| | Val-A | 60 | 48 | 1·9 | 1·1 | 0·9 | 0·6 | 0·9 |
| Creatine phosphokinase | M-I | 39 | 45 | 7·6 | 1·3 | 1·5 | 1·8 | 1·5 |
| (U/l) | M-II | 140 | 133 | 7·4 | 2·2 | 1·7 | 2·4 | 2·2 |
| | Val-A | 148 | 136 | 3·9 | 2·6 | 0·8 | 2·4 | 2·4 |
| γ-glutamyl transferase new | M-I | 14 | 12 | 6·5 | 1·3 | 1·5 | 1·6 | 1·5 |
| (U/l) | M-II | 59 | 51 | 4·4 | 1·5 | 2·5 | 0·0 | 1·5 |
| | Val-A | 61 | 52 | 1·4 | 3·0 | 0·6 | 1·2 | 1·2 |
| Glutamate dehydrogenase | M-I | 4·7 | 4 | 14·4 | 8·2 | 11·6 | 10·1 | 10·1 |
| (U/l) | M-II | 11·5 | 11 | 8·8 | 4·2 | 7·1 | 5·3 | 5·3 |
| | Val-A | 17·3 | 16 | 5·2 | 4·0 | 8·3 | 4·0 | 4·0 |
| Lactate dehydrogenase | M-I | 181 | 167 | 4·1 | 0·4 | 1·5 | 0·4 | 0·4 |
| (U/l) | M-II | 393 | 358 | 3·2 | 1·0 | 0·6 | 0·3 | 0·6 |
| | Val-A | 313 | 289 | 1·7 | 0·5 | 0·5 | 0·5 | 0·6 |
| Lipase | M-I | 388 | 396 | 5·0 | 1·1 | 0·9 | 1·7 | 1·1 |
| (U/l) | M-II | 695 | 702 | 3·2 | 1·8 | 1·6 | 1·4 | 1·6 |
| | Val-A | 303 | 317 | 2·2 | 0·9 | 0·9 | 0·8 | 0·9 |
| *2. Flame photometry* | | | | | | | | |
| Sodium | M-I | 139 | 140 | 2·3 | 0·4 | 0·0 | 1·0 | 0·4 |
| (mmol/l) | M-II | 119 | 117 | 2·0 | 0·0 | 0·0 | 0·2 | 0·0 |
| | Val-A | 124 | 124 | 2·6 | 1·1 | 0·5 | 1·2 | 1·1 |
| Potassium | M-I | 4·3 | 4·3 | 2·2 | 0·0 | 0·0 | 1·9 | 0·0 |
| (mmol/l) | M-II | 6·7 | 7·2 | 3·0 | 0·7 | 1·1 | 3·2 | 1·1 |
| | Val-A | 7·5 | 6·9 | 3·5 | 0·9 | 0·7 | 2·1 | 0·9 |
| *3. Substrates* | | | | | | | | |
| Blood urea nitrogen | M-I | 5·2 | 5 | 2·0 | 0·6 | 1·3 | 0·5 | 0·6 |
| (mmol/l) | M-II | 17·3 | 18 | 2·0 | 1·1 | 0·7 | 0·7 | 0·7 |
| | Val-A | 18·9 | 17 | 4·6 | 0·4 | 0·4 | 0·5 | 0·4 |
| Bilirubin | M-I | 22·1 | 22 | 6·4 | 1·3 | 0·7 | 2·4 | 1·3 |
| (umol/l) | M-II | 70 | 84 | 3·2 | 2·9 | 0·0 | 4·6 | 2·9 |
| | Val-A | 74 | 76 | 4·0 | 1·9 | 1·4 | 0·0 | 1·4 |
| Calcium | M-I | 2·3 | 2·2 | 1·9 | 0·7 | 0·7 | 1·3 | 0·7 |
| (mmol/l) | M-II | 3·2 | 3·2 | 1·7 | 0·0 | 2·2 | 1·0 | 1·0 |
| | Val-A | 3·4 | 3·0 | 2·2 | 1·6 | 0·9 | 0·7 | 0·9 |
| Cholesterol | M-I | 4·6 | 4·4 | 2·0 | 0·4 | 0·7 | 0·7 | 0·7 |
| (mmol/l) | M-II | 3·0 | 2·8 | 2·0 | 0·6 | 0·5 | 0·5 | 0·5 |
| | Val-A | 2·3 | 2·4 | 2·0 | 0·9 | 0·6 | 0·4 | 0·6 |
| Creatinine | M-I | 112 | 113 | 3·9 | 0·7 | 0·8 | 0·7 | 0·7 |
| (umol/l) | M-II | 481 | 511 | 2·5 | 0·0 | 0·0 | 2·3 | 0·0 |
| | Val-A | 265 | 307 | 2·4 | 1·3 | 1·2 | 0·0 | 1·2 |
| Phosphate | M-I | 0·9 | 0·8 | 3·8 | 1·2 | 0·8 | 0·9 | 0·9 |
| (mmol/l) | M-II | 2·1 | 1·9 | 2·8 | 2.0 | 1·6 | 1·8 | 1·8 |
| | Val-A | 2·6 | 2·4 | 2·5 | 0·8 | 0·9 | 0·9 | 0·9 |
| Iron | M-I | 29 | 28·0 | 2·4 | 1·2 | 1·2 | 1·8 | 1·2 |
| (umol/l) | M-II | 44 | 42·0 | 3·5 | 1·8 | 1·6 | 1·1 | 1·6 |
| | Val-A | 13 | 12·5 | 2·5 | 2·6 | 2·7 | 1·0 | 2·6 |

*Table 6—continued.*

| Analyte (Units) | Control material | Assigned values | Found value | Between days | Imprecision Within-run (Three series) | | | Median |
|---|---|---|---|---|---|---|---|---|
| Magnesium | M-I | 0·95 | 1·0 | 4·9 | 0·8 | 5·1 | 1·4 | 1·4 |
| (mmol/l) | M-II | 1·96 | 1·6 | 4·4 | 1·6 | 3·6 | 1·0 | 1·6 |
| | Val-A | 0·93 | 1·8 | 3·7 | 0·9 | 2·6 | 0·9 | 0·9 |
| Total protein | M-I | 67 | 64 | 3·5 | 1·0 | 1·2 | 0·8 | 1·0 |
| (mmol/l) | M-II | 48 | 44 | 3·2 | 0·7 | 0·0 | 1·1 | 0·7 |
| | Val-A | 51 | 44 | 3·4 | 0·0 | 0·5 | 0·9 | 0·5 |
| Triglycerides | M-I | 0·96 | 0·9 | 2·8 | 1·0 | 0·6 | 1·6 | 1·0 |
| (mmol/l) | M-II | 1·97 | 2·1 | 2·6 | 1·4 | 1·0 | 1·7 | 1·4 |
| | Val-A | 0·6 | 0·8 | 3·3 | 1·0 | 1·1 | 0·9 | 1·0 |
| Uric acid | M-I | 305 | 299 | 1·9 | 0·7 | 0·5 | 0·9 | 0·7 |
| (umol/l) | M-II | 498 | 534 | 2·2 | 0·8 | 1·1 | 0·4 | 0·8 |

*Table 7. Linearity of the enzyme and substrate tests.*

| | Unit | Measured up to | Found linear up to | Upper limit of linearity (declaration of manufacturer) |
|---|---|---|---|---|
| *Enzymes* | | | | |
| Alkaline phosphatase | U/l | 5200 | 5200 | 1800 |
| Alanine aminotransferase | U/l | 1930 | 1930 | 400 |
| Amylase | U/l | 558 | 558 | 430 |
| Aspartate aminotransferase | U/l | 880 | 880 | 400 |
| Creatine kinase | U/l | 800 | 800 | 1100 |
| γ-glutamyltransferase | U/l | 2100 | 1790 | 1600 |
| Glutamate lact. dehydrogeanse | U/l | 320 | 150 | 38 |
| Lactate dehydrogenase | U/l | 670 | 670 | 650 |
| Lipase | U/l | 1200 | 650 | 700 (25 °C) |
| *Substrates* | | | | |
| Blood urea nitrogen | mmol/l | 44 | 44 | 33 |
| Bilirubin | μmol/l | 430 | 430 | 390 |
| Calcium | mmol/l | 6·5 | 6·5 | 5·1 |
| Cholesterol | mmol/l | 17·2 | 17·2 | 15·5 |
| Creatinine | μmol/l | 2840 | 2840 | 2650 |
| Inorganic phosphate | mmol/l | 6·9 | 6·9 | 6·5 |
| Iron | μmol/l | 215 | 215 | 180 |
| Magnesium | mmol/l | 2·0 | 2·0 | 2·0 |
| Total protein | g/l | 150 | 150 | 110 |
| Triglycerides | mmol/l | 15·9 | 15·9 | 6·8 |
| Uric acid | μmol/l | 1770 | 1770 | 1190 |

course of temperature lowering caused by the addition of cooled reagent 1 showed that the reaction temperature of 37 °C had been reached in the cuvette before reading by photometer 1 and additon of reagent 2 (168 and 204 s later, respectively). This is illustrated in figure 1.

### *Imprecision*

The within-run imprecision and the between-day imprecision are shown in figure 2(*a*) (enzymes) and figure 2(*b*) (substrates). For two of the control sera the imprecision values are also given for the comparison instruments. The values presented in the figures are medians from three independent measurement series. Exact figures are listed in table 6.

It is apparent from this data that 15 of the 22 evaluated tests gave a within-run imprecision of below 1·5%. Six of the seven remaining tests gave values below 2·9% – only glutamate dehydrogenase gave a high imprecision which, however, is not due to a failure of the test or of the analyser, but is a consequence of the low activities which are measured by this test. This performance can be rated as very good.

The between-day imprecision was below 4·5% for at least two out of three control sera. Exceptions were creatine

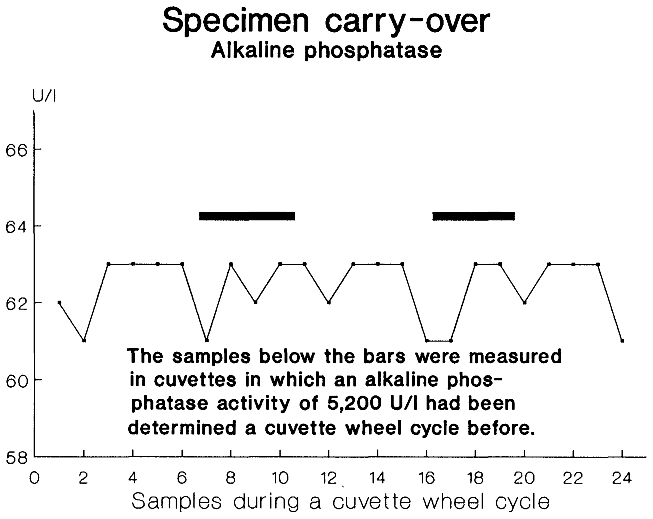

*Figure 4. Specimen-related carry-over: alkaline phosphatase measurements of 24 samples (one cycle of the cuvette wheel) of a serum pool with low analyte activity. Samples with an activity of alkaline phosphatase of 5200 U/l had been measured in cuvettes 6 to 10 and 16 to 20, respectively, during the preceeding wheel cycle.*

kinase (7·6, 7·2 and 2·9%) and, again, glutamate dehydrogenase.

The between-day imprecisions of the comparison instrument are also shown in figure 2 (for Monitrol I and II only) – the AU 5031 matches this performance.

Close imprecisions were reported by other evaluators who found values surpassing the comparison instrument [3].

### *Drift*

The results of investigations on drift over 8 h were inspected visually after blotting the percentage deviations from the starting value (100%) over a time scale. Figure 3 displays three representative plots. A continuous trend either increasing or decreasing could not be detected for any of the 22 parameters. Deviations were below 5% for all tests. This result is in contrast to Rohac *et al.* who reported drifts for cholesterol, triglycerides and uric acid, but did not discuss this finding further [3]. Hodgin *et al.* omitted this aspect from their evaluation [2].

### *Linearities*

Both the linearities found, and the linearity ranges declared by the manufacturer, are given in table 7. The linearities found were better than the manufacturer's for all tests with the exceptions of creatinine kinase and lipase. In fact, a limited linearity was found only for gamma-GT, glutamate dehydrogenase and lipase. For the remaining tests the limitation of linearity is beyond the highest value measured. The findings are in good agreement with the results of other evaluators [2 and 3].

### *Specimen-related carry-over*

A potential carry-over of sample residue due to an insufficient washing of the cuvettes was investigated by measuring alkaline phosphatase in cuvettes which had been used before for the determination of the same analyte in a very high activity (5200 U/l). The results were compared with those which were obtained from the same sample in cuvettes, which, a cuvette wheel before, had been used for the determination of this analyte in a low activity (62 U/l). The results are displayed in figure 4 which shows the measured values of this sample in 24 cuvettes (one cycle of the cuvette wheel). It is apparent from the figure that no difference could be detected. The Wilcoxon test did not show a significance difference between both sets of values ($p$ = 0·84). Thus, the specimen carry-over was smaller than 0·04%. Carry-over effects between 0·1% and 5·33% were reported for different parameters by other evaluators [1 and 2]. However, neither investigator took into consideration the sequence of cuvettes which were assigned to the respective tests.

### *Specimen-independent carry-over*

A carry-over caused by transfer of reagent due to an insufficient washing of the mixer blades was tested in two reagent lines which were served by the same mixer blade (reagent line 1 and reagent line 5). The triglycerides values (means of quadruplicate measurement in reagent line 5) in 10 serum samples did not differ in the Wilcoxon test ($p$ = 0·76), regardless of whether concomitant lipase determination had been carried out in reagent line 2 or not. Rohac *et al.* carried out a similar experiment and found a considerable carry-over leading to threefold lipase measurements in combination with triglycerides. Their results, however, are difficult to discuss because of a lack of experimental detail [3].

### *Assessment of accuracy*

#### *Recovery of assigned values in quality-control sera*

The recovered values of all analytes in 10 commercial control sera were expressed as percentage of the assigned values. The medians and the 10th and 90th percentiles of the respective values are displayed in figure 5. Fourteen of 22 tests yielded recoveries within the 5% range. Five tests were in the range between 5% and 10% (γ-glutamyl transferase, lactate dehydrogenase, bilirubin, phosphate and total protein) and three tests were in the range between 10 and 18% (alanine aminotrans-ferase, aspartate aminotransferase and creatinine).

#### *Comparison with other analytical routine procedures*

The results obtained by determination of all parameters in 100 routine sera on the Olympus AU 5031 and on routine instruments (see tables 3 and 4) were correlated using the method of Bablock and Passing [4]. The results of this statistical evaluation are given in table 8 and figure 6.

The correlations were close for all tests. A correction was unnecessary for eight out of 22 tests (γ-glutamyl transferase, sodium, blood urea nitrogen, cholesterol, creatinine,

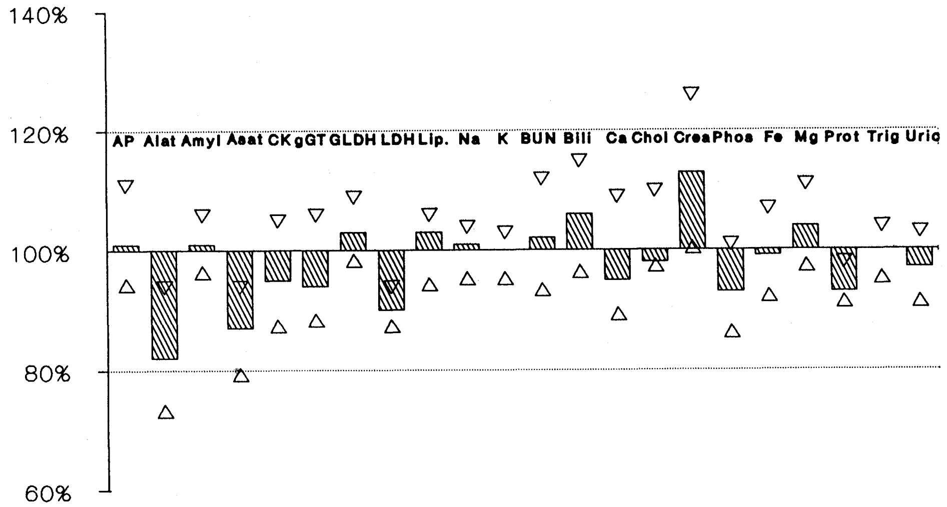

*Figure 5. Recovery of assigned values in 12 commercial control sera. The columns represent medians and percentiles (10th, 90th) of the percentage deviations from the assigned values.*

iron, triglycerides). The remaining 14 tests differed either in slope (bilirubin, potassium), or in intercept (alanine aminotransferase, creatinine phosphokinase, glutamate dehydrogenase, lipase, inorganic phosphate, uric acid and calcium), or both (alkaline phosphatase, amylase, aspartate aminotransferase, magnesium and total protein). Statistical data are given in table 8, which displays slope, intercept and the correlation coefficient after regression analysis according to the equation: $y = a * x + b$ where $y$ = the AU 5031 and $x$ = the comparison instrument. The reasons for the discrepancies were various: different physiochemical methods on the comparison instruments (see tables 3 and 4), different analysis temperatures (see the section on Reagents and comparison instruments) and a lack of accuracy for some methods on the AU 5031 (see figure 5). However, it is unlikely that these discrepancies can be attributed to the performance of the analyser but arise because of the methodological discrepancies summarized above.

The results of these comparisons were discussed with the manufacturers of the respective tests. In consequence, factors of alkaline phosphatase and lactate dehydrogenase have been corrected in accordance with data from the laboratories evaluating the AU analyses.

*Assessment of practicability*

Despite the size and capacity of the analyser, the Olympus AU 5031 is easy to run. It is easily accessible from all sides and all mechanical movements can be seen. The data-processing software is sophisticated but can be mastered by a technician after a training period of a few days. The software contains an extensive diagnostic program, which allows a specific detection of technical or electronic problems. Together with a complete system of LED controls of all mechanical movements problems can rapidly be tracked to their origin thus allowing immediate remedy or better communication with the Olympus service.

The preanalytical preparation of the analyser consists of four steps, which require the time periods given in parentheses: warming up to 37 °C (60 min); washing of cuvettes and filling of reagent lines with reagent (20 min); reagent blanking plus running controls (20 min). In order to circumvent the long warm-up phase the authors use an electronic clock which starts the warming-up automatically before routine work begins. Thus, the preanalytical phase is reduced to 40 min and this time is required for such routine activities as checking and replacing the reagents.

During the evaluation period and the subsequent routine work no total shut down of the analyser occurred. However, during the routine period a single unit broke down twice: in both cases the reason was a faulty adjustment of the sensors which control the movement of the washing units. After correction in all three units this problem did not occur again. The most practical and most rapid solution for this emergency was to concentrate 'vital' tests on the remaining units. It was easier to transfer reagents and programs from the faulty unit to the other units, than to exchange the total unit as mentioned above ('Description of the AU 5031 analyser'). This transfer of tests was completed in less than 30 min, including washing of the reagent lines. The units were repaired by the Olympus service technicians before the next morning. No other breakdown causing a severe delay occurred thereafter.

Two modes of sample identification are possible: via terminal or via bar-code identification. The manual prescribes a correct positioning of the bar code onto the sample cup; the vertical slant should be lower than 5°.

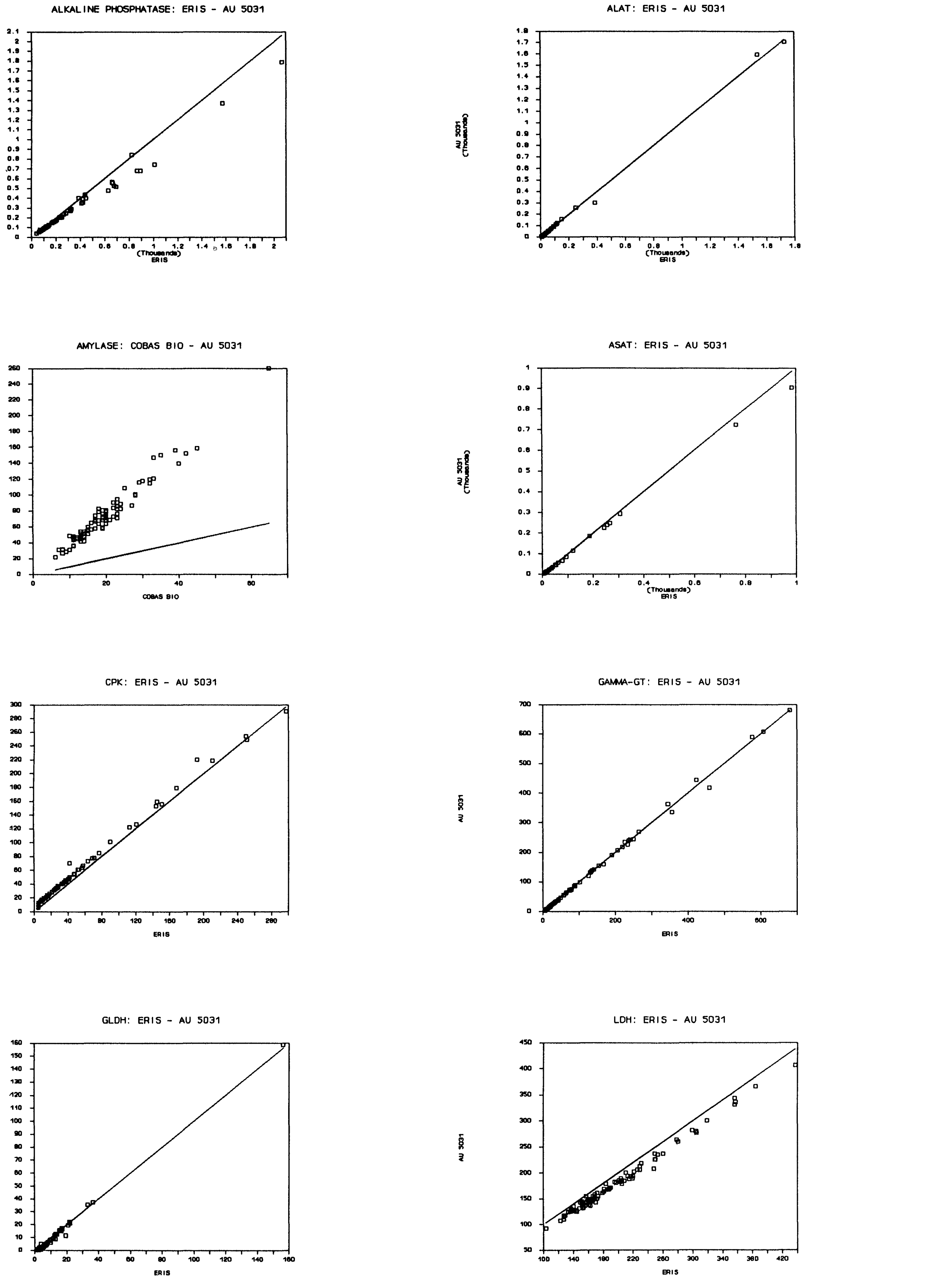

*Figure 6(a). Graphical plots of instrument comparison. Statistical data are listed in table 8.*

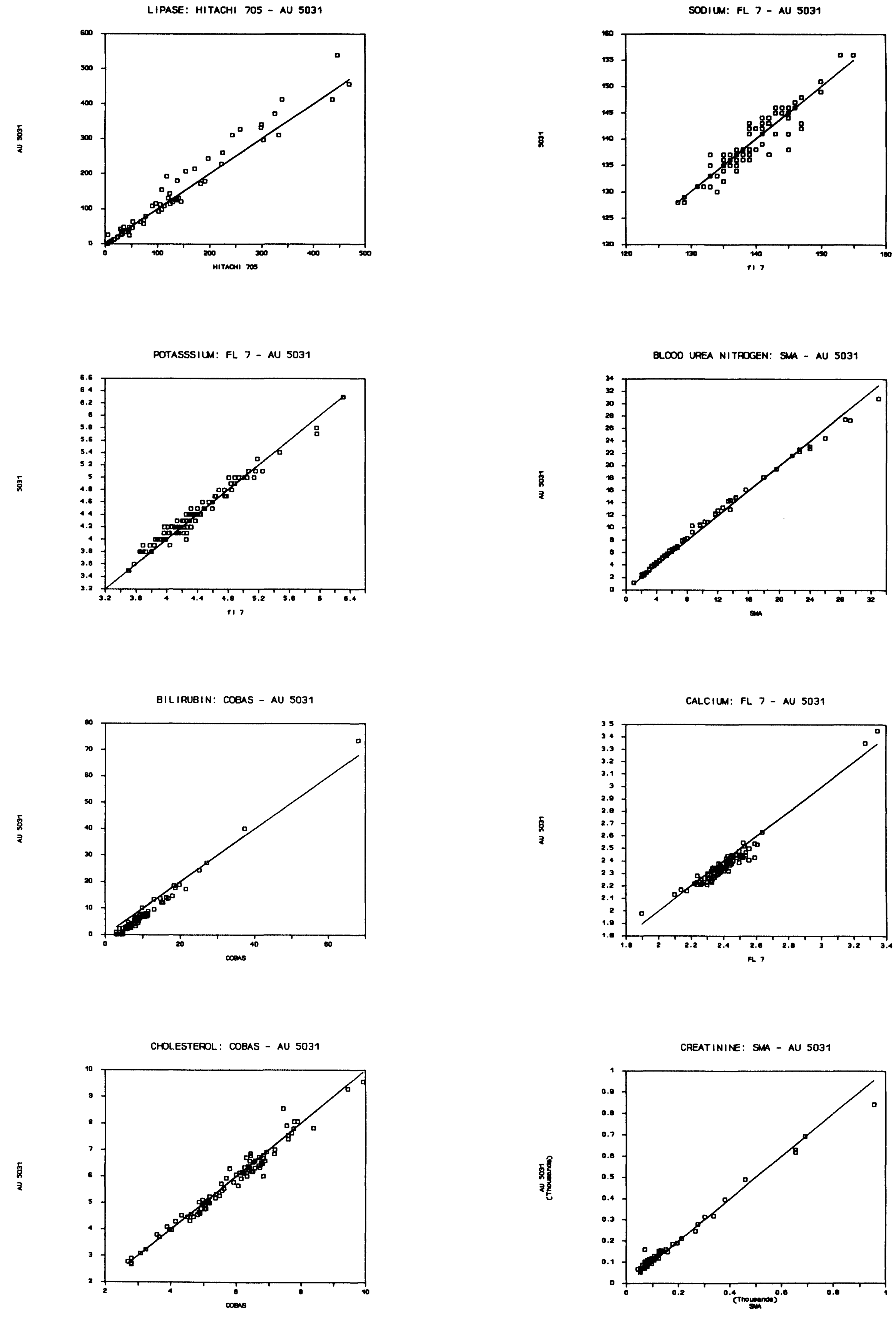

*Figure 6(b). Graphical plots of instrument comparison. Statistical data are listed in table 8.*

However, angles up to 15° are read correctly. In addition, the vertical position may vary up to 10 mm.

The following points would improve the performance of the analyser.

(1) Changing from bar-code reading to sample identification via the terminal required a complete termination of bar-code analyses. This causes inevitable delays of 20 min for each change.

(2) The software which produces the print-out is inflexible. For example, all available tests and references ranges are printed, even if only one test has been required for a given sample. In addition, flagging of pathologic values ('high' or 'low') cannot be defined differently for both sexes. As a result, this flag is set only according to one reference range, either female or male. Finally, the matrix printer which is delivered with the analyser is slow and noisy. The authors have connected a personal computer which runs a versatile custom-made report software and serves a quiet, and very rapid, ink-jet printer (the Epson SQ 2500).

(3) The tubing system by which the serum sample is transported to the flame photometer consists of three parts: two narrow tubes made of stainless steel and silicone tubing. Frequent blocks in this system were encountered, which were caused by tiny clots which either pre-existed in the sample or resulted from delayed clotting which could have been triggered by contact with the materials described above. Since an obstruction in the tubing system can result in time losses of up to 45 min this system should be replaced by a continuous Teflon tubing.

These suggestions have been put to Olympus and it appears that all three are being tested.

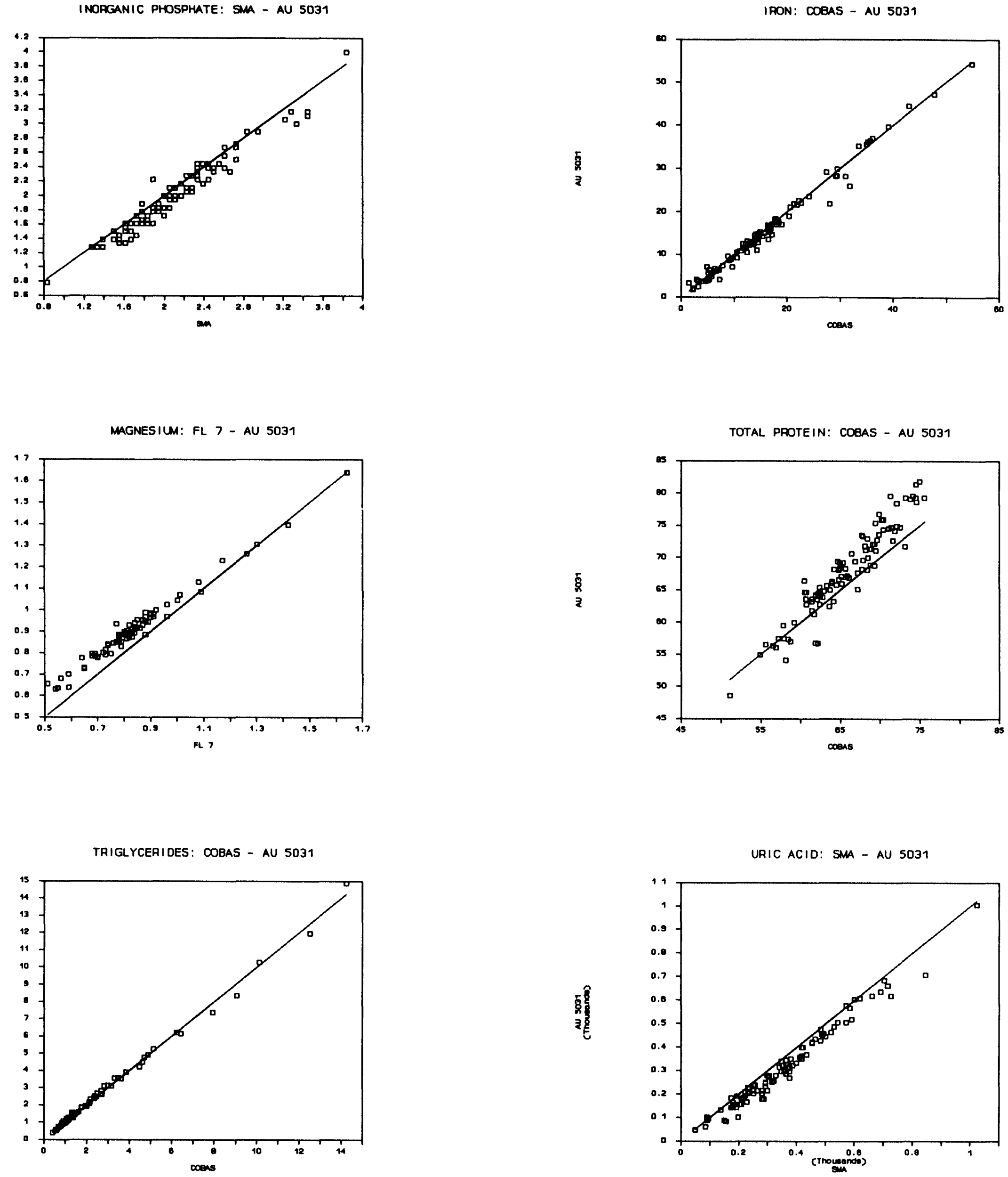

*Figure 6(c). Graphical plots of instrument comparison. Statistical data are listed in table 8.*

*Table 8. Comparison with routine instruments.*

| Method | Regression analysis ($y$ = a*$x$ + b)<br>$y$ = AU 5031; $x$ = comparison instrument<br>SE = standard error of the slope<br>CC = correlation coefficient | | | | |
|---|---|---|---|---|---|
| | *N* | a | SE | b | CC |
| *1. Enzymes* | | | | | |
| Alkaline phosphatase | 100 | 0·845 | 0·013 | 5·5 | 0·994 |
| Alanine aminotransferase | 100 | 1·051 | 0·004 | −2·6 | 0·999 |
| Amylase | 100 | 3·925 | 0·005 | −4·0 | 0·980 |
| Aspartate aminotransferase | 100 | 0·932 | 0·002 | −1·5 | 0·999 |
| Creatin kinase | 100 | 1·000 | 0·005 | 8·0 | 0·999 |
| γ-glutamyltransferase | 100 | 1·000 | 0·004 | −1·0 | 0·999 |
| Glutamate lact. dehydrogenase | 100 | 1·012 | 0·008 | −1·2 | 0·998 |
| Lactate dehydrogenase | 100 | 0·972 | 0·010 | −7·1 | 0·996 |
| Lipase | 100 | 1·062 | 0·011 | 6·6 | 0·982 |
| *2. Flame photometry* | | | | | |
| Sodium | 100 | 1·002 | 0·032 | 0·7 | 0·948 |
| Potassium | 100 | 0·993 | 0·017 | 0·1 | 0·986 |
| *3. Substrates* | | | | | |
| Blood urea nitrogen | 100 | 1·00 | 0·007 | 0·3 | 0·998 |
| Bilirubin | 100 | 1·17 | 0·009 | 3·4 | 0·994 |
| Calcium | 100 | 0·99 | 0·040 | −0·1 | 0·942 |
| Cholesterol | 100 | 0·98 | 0·019 | 0·0 | 0·985 |
| Creatinine | 100 | 0·97 | 0·013 | 18 | 0·994 |
| Inorganic phosphate | 100 | 1·00 | 0·023 | −0·1 | 0·976 |
| Iron | 100 | 1·00 | 0·013 | 0·8 | 0·984 |
| Magnesium | 100 | 0·97 | 0·012 | 0·2 | 0·989 |
| Total protein | 100 | 1·24 | 0·024 | −19 | 0·951 |
| Triglycerides | 100 | 1·02 | 0·007 | 0·0 | 0·998 |
| Uric acid | 100 | 0·97 | 0·017 | −24 | 0·988 |

### Conclusion

The Olympus AU 5031 is a powerful analyser which can be tailored according to the needs of medium and large laboratories. During the evaluation and the subsequent routine period the analyser proved to be very reliable. Both the photometer linearity in a unit and rapid temperature equilibration upon addition of cooled reagent were found satisfactory. Imprecision values very good, while the accuracy of some tests required corrections. Correlations with other routine analysers was close. Neither drift nor a carry-over were detected. Finally, due to the wide ranges of most tests, only a few samples needed to be re-run.

### References

1. Haeckel, R., Busch, E. W., Jennings, R. D., Kokholm, G. and Truchaud, A., *ECCLS Document*, Vol. 3, No. 2 (1986).
2. Hodgin, P. B. Jr., Moore, W. F. III and Kincaid, H. L., *Journal of Automatic Chemistry*, **10** (2) (1988), 79.
3. Rohac, M., Hotschek, H., Hajdusich, P., Bayer, P. M., Fischer, G. and Gabl, F., *Österreichische Krankenhauszeitung*, **29** (1988), 315.
4. Passing, H. and Bablock, W. J., *Clinical Chemistry and Clinical Biochemistry*, **22** (1984), 431.

